# *‘Even if you are guilty, you’re still a human being’:* the impact of stigma on dignity and quality of healthcare from the perspective of people detained in police custody in England

**DOI:** 10.1186/s40352-026-00444-x

**Published:** 2026-08-04

**Authors:** Michelle Addison, Stephanie Mulrine, Iain McKinnon, Gethin Rees

**Affiliations:** 1 Department of Sociology, Durham University, Durham, United Kingdom; 2 School of Medicine, University of Sunderland, Sunderland, United Kingdom; 3 Population Health Sciences Institute, Newcastle University, Newcastle upon Tyne, United Kingdom; 4 School of Geography, Politics and Sociology, Newcastle University, Newcastle upon Tyne, United Kingdom

**Keywords:** Stigma, Police custody, Dignity, Health, Inequality

## Abstract

**Background:**

The aim of this paper is to explore harms to health and dignity generated through stigma from the perspective of people detained in police custody, a neglected population in healthcare research. We adopt an original theoretical approach, bringing together a human rights lens to health, stigma, and the Inverse Care Law (ICL) to show how stigma contributes to the degradation of dignity and poor experiences of *quality* care within custody.

**Methods:**

The study utilises qualitative semi-structured interviews with 41 people with experience of being detained in police custody, across two police constabulary locations (North and South England). Data were analysed thematically using NVivo qualitative software.

**Results:**

Detainees experienced stigma whilst in police custody because of identity characteristics, status, drug use, mental health, and frequent healthcare requests. Stigmatising and dehumanising interactions with detention officers (DO) and Healthcare Practitioners (HCPs) align with harms to health and wellbeing, and contributed to a degradation of dignity amongst detainees.

**Conclusions:**

This novel research reveals that the impact of stigma in police custody settings materialise as harms to health and wellbeing, and poorer quality healthcare. We argue that stigma interactions in custody settings undermine a human rights approach to health specified in the AAAQ standards set out by the World Health Organisation, and also reproduce the inverse care law. As part of a wider recommended overhaul of police custody in England and Wales, we advise this should include urgent anti-stigma work in police custody settings to address health inequities across delivery and provision of good *quality* health care.

## Background

Police custody is a highly pressurised environment that is often compared to a hospital accident and emergency ward (National Police Chiefs' Council (NPCC) 2022). A range of factors make custody a volatile space—including the unpredictability of detainees’ health needs and behaviours, the frequency of turnover in cells, operational peaks generating a high ratio of detainees to only one onsite health care practitioner (or none at all), and workload pressure on an already stretched custody team comprising the custody sergeant (CS), and detention officers (DO) (Rees et, al. 2026; Stockdale et, al. 2022). These factors can range in intensity depending on geographical location (rural/inner city) and available resources, meanwhile serving as a barometer for how well the system is responding to demand and mitigating risk on any given day. Nevertheless, managing and controlling for risk by doing the *‘right things at the right time’* is vital to ensure safe and effective operations in the custody suite (College of Policing, 2025; (National Police Chiefs' Council (NPCC), 2022:13). This can generate tension between the provision of care and the management of risk, particularly for healthcare practitioners (HCPs) who are trained general nurses (often) employed by private companies (two police forces in England employ HCPs directly) and are embedded into police custody suites undertaking 12-h shifts.

The impacts of the ‘care-custody’ paradox as it is framed have been variously discussed by the authors – Rees found that HCPs were able to find ways to synthesise their medical and legal responsibilities to attend to both immediate care (medication provision, attending to wounds, generally not therapeutic) and risk, by focusing on a bio-medical model of health need (Rees, 2023); likewise, the research Addison conducted around brief interventions in custody showed that delivery of care in this setting was complicated by a custody culture focused on containment, risk management, and perceptions of ‘deservingness’ (Addison et, al. 2018). Similarly, McKinnon and Finch’s (2018) qualitative study looking at the design and delivery of health screening risk assessments in police custody illustrates the paradoxical nature that can arise across care delivery and risk management.

These tensions around ‘care’ and ‘risk’ in custody are important to consider because the healthcare that is received by detainees in police custody, a neglected population in healthcare research, can influence health outcomes and potentially widen health inequalities (Wardrop et al., 2021). This can have ramifications on wider health and social care systems in the community by heightening pressure and demand through exacerbating rather than reducing a person’s complex needs upon release from custody. This is evidenced in Wardrop et al.’s. scoping review of 37 international studies using a range of study designs, which shows that pre-existing health needs of detainees can often go unrecognised, and the delivery of care in custody tends to focus on a bio-medical model of disease and co-morbidity at the expense of a more person-centred approach to health and wellbeing. Wardrop et, al. identified care provision as particularly important to develop as part of the HCP role, addressing both the wider social determinants of health and potentially reducing re-offending behaviours (2021). Whilst having basic and mundane health needs met is crucial to managing risk in custody (Rees, 2023), it is not enough. As such, exploring how care is experienced by detainees in custody is important because poor quality care can have negative consequences for health outcomes. In this paper we explore harms to health and dignity generated through stigma from the perspective of people detained in police custody. In this paper we bring together different bodies of literature across Criminology, Sociology and public health: we draw on a human rights approach to health and the Inverse Care Law (ICL) to make an original theoretical contribution to knowledge, arguing that stigma contributes to the degradation of dignity and inverse experiences of *quality* care within police custody.

According to Home Office statistics for 2024 in England and Wales, 901,758 people were detained in police custody (7% of these were children), and 79% of these detainees were only detained once in that year (Home Office, 2024). Of those detained, the majority were male (84%); 78% were of a white ethnic background (excluding Metropolitan police service) and were mainly aged between 21–40 years old. A third of offences were for violence against the person (34%), followed by non-notifiable offences (29%), theft (12%) and drug offences (8%). Approximately 7% of adult male detainees were identified as vulnerable and this was 10% for adult female detainees. although these figures are contested by the National Appropriate Adult Network (NAAN) who cite closer to 39% of adults in police custody have a mental disorder (Bath 2020); Home Office, 2024). Overall, this data about detainees begins to build a picture of a subpopulation who have become entangled with the criminal justice system and broadly present with complex behaviours, compounding health and social care needs, and challenging social contexts (National Police Chiefs' Council (NPCC), 2022); Samele et al., 2021). Whilst recidivism is an urgent societal and political concern National Police Chiefs' Council (NPCC) 2022), we should also be mindful that at least a fifth of those detained in 2024 have experienced the compounding negative health effects and disruption to ordinary life on *multiple* occasions as a result of detention (Home Office, 2024). Therefore, experiences of care in police custody should be a priority public health concern (Independent Office for Police Conduct, 2026).

The rights, entitlements and welfare of all detainees in custody in England and Wales are protected by the Police and Criminal Evidence Act 1984 (PACE) Code of Practice C, which sets out the statutory framework that must be adhered to by all professionals working in the custody suite at all times (HM Government, 2019). Human rights are also clearly defined by the World Health Organisation (WHO) where the ‘right to health is indivisible from other human rights’ (World Health Organization, 2025). The WHO set out core standards regarding the human right to health, which include the 1966 United Nations (UN) International Covenant on Economic, Social and Culture Rights AAAQ framework: availability, accessibility, acceptability, and quality (AAAQ standards) (United Nations Human Rights: Office of the High Commissioner, 1966; World Health Organization). Of interest here are the key components of ‘quality’ healthcare – the WHO state that quality care should be safe, effective (meeting health needs), people-centred (providing care that is responsive to need), timely, equitable (care that does not vary in quality on account of status), integrated and efficient (maximises benefit) (World Health Organization). This human rights approach to healthcare is inherent in the PACE statutory framework and has been discussed elsewhere by scholars focused on detention in prison custody in the US and UK (Exworthy et al., 2012) (Exworthy et al., 2011) (Rees et, al. 2026).

Despite the rights envisaged under PACE, the *experience* of quality health*care* for detainees is, arguably, less of a priority for custody and healthcare staff when set against more urgent and immediate indicators of risk, which are considered to be managed and controlled for in police custody. The College of Policing states that, in addition to the efficient running of operational duties like maintaining custody records, the physical condition of the custody suite, review times and sufficient staffing levels, professionals must ensure that *‘all risks, vulnerabilities and welfare needs of detainees are being adequately managed’* (College of Policing, 2025, n.p.). In response to this mandate, staff are taught to recognise behavioural signs of distress amongst detainees as markers of increased risk – where critical risk leads to a death in custody. A death in police custody, or following detention, is classed as a serious incident and a matter of public concern (Independent Office for Police Conduct, 2024b). An incident of this nature prompts an investigation by the Independent Office for Police Conduct (IOPC). Managing and controlling risk in police custody is absolutely core to strategic planning and day-to-day operations.

Recently, the *National Strategy for Police Custody* set out by the National Police Chiefs’ Council (NPCC) recognises that police custody has been overly focused on risk management (National Police Chiefs' Council (NPCC), 2022) leading to detrimental impacts on detainees (Skinns, 2019). This underlines the timeliness of our own study. This national strategy attempts to drive forward improvements to how risk is framed, managed and controlled within custody (e.g. Custody Early Warning Score – CEWS, and pre-release risk assessments; see Lyall et al., 2022) via six strategic principles^1^ and four thematic work areas. In particular, the strategy attempts to foreground ‘care’ and identifies people in custody as *‘some of the most challenging and vulnerable in society’* (2022: 1). It is also worth noting here that detainees meet the criteria of a marginalised and vulnerable population with multiple complex health and social care needs outlined by the NHS priority health agenda—CORE20PLUS5 (NHS England, 2022), not least because they have had their liberty taken away. This means that in police custody people who are detained no longer have agency to access a healthcare provider or their regular medications, instead this is determined by the Custody Sergeant and the HCP.

Whilst the primary focus of detention in custody is to facilitate a criminal investigation, the NPCC strategy also aims to ensure that a detainee’s dignity is protected and that they leave custody *‘in a better condition or circumstances than when they arrived’*; the NPCC add that custody should be *‘as far as is possible to the long-term benefit of the detained person’* (National Police Chiefs' Council (NPCC), 2022: 7) by linking detainees up to a range of health and social care organisations.

However, this framing of dignity in the National Police Custody Strategy is ambiguous and open to interpretation; as Skinns et al. note, when dignity is not fully thought through this can mean that less obvious harms that lead to humiliation and dehumanisation may be missed, concealed, overlooked or invisible (Skinns et al., 2020). What do the NPCC mean by *dignity*? At present, this is unclear in their police custody strategy; our sense is that this could be strengthened through co-production methods involving people with lived experience and the academic community. For instance, in Skinns et, al.’s (2020) mixed methods study, they examined factors that influenced experiences of dignity in police custody from the perspective of justice involved individuals – they identified dignity as meaning being treated as an equal because of a shared status as *human beings* (our emphasis added). Drawing on survey data from n-371 detainees across 27 custody facilities in 13 police forces in England and Wales, their research showed that being treated with compassion and respect, and gestures like a chat and a cup of tea, and also having basic material needs met, fostered a sense of dignity amongst detainees (Skinns et al., 2024). That said, these experiences of dignity are intersectional—young adults and Black, Asian and Minority Ethnic detainees were less likely to feel they had been treated with dignity, and these individuals were also less trusting of accountability mechanisms (Skinns et al., 2020). Skinns et al., (2020:1667) describe dignity harms as material and relational, and they helpfully draw attention to the European Court of Human Rights (ECtHR) Article 3 identification of failures to maintain dignity, which can include: ‘*excessive coercion and threatened coercion, poor material conditions and inadequate access to health or mental health care’* (Council of Europe, 1950, Art. 3). Of particular interest to our own study is where Skinns’ et al. note that ‘degrading treatment’ denotes a failure to maintain a person’s dignity under Article 3 of the convention. We seek to unpack this notion of degrading treatment in police custody through a relational lens of stigma. We are interested in how interactions can be experienced as stigmatising and dehumanising, from the perspective of people who are detained in police custody, and how these experiences impact health and wellbeing. We illustrate harms to health generated through stigma (Addison et, al. 2023, 2025; Addison, 2023). Finally, we draw on the *inverse care law* and AAAQ standards to show that degradation of dignity can be aligned with inverse experiences of *quality* care within custody (Tudor Hart, 1971; World Health Organization, 2025).

### Theoretical framework

Stigma can be present at macro and micro levels in society simultaneously and relationally: for instance, in interactions between people, structurally embedded across policies, politics and service design, it can be inscribed symbolically to place and space (Wacquant, 2008), read off bodies and supposed ‘deviant’ practices (Addison, 2023), function as symbolic violence and misrecognition (Fraser, 2001); stigma can be anticipated and internalised as shame ‘under the skin’ (Addison 2023; Addison et, al. 2022; Corrigan, 2022; Dolezal, 2022), and weaponised to accrue and protect power differences (Scambler, 2018; Tyler, 2020). Stigmatisation can serve as a form of social control – Link and Phelan describe it as a way of keeping someone ‘down’ (exploited), ‘in’ (compliant), and ‘away’ (socially excluded) (Link & Phelan, 2001). Stigma is not static or universally experienced – it can mutate like a virus over time, and it can vary in strength, intensity and stickiness – a person may not always be stigmatised or passively victimised by stigma harms (Farrimond, 2023). Indeed, the effects of stigma can reduce, it can be ignored, subverted, reclaimed, disregarded and resisted (Farrimond, 2023; Thomas et al., 2025) depending on changes in cultural values, context and cultural capital.

All things considered, stigma is complex. It is very often invisible to us and indeed, unintentionally inflicted at times. However, the commonality across these models, definitions and experiences is that stigma is fundamentally an act of harm which has the potential to be damaging and degrading to the subject – albeit to different degrees and depths (occasionally leaving no visible ‘mark’), despite the many ways and reasons in which it can be inflicted, resisted or reclaimed in potentially positive ways (Addison et, al. 2022; Thomas et al., 2025). It is vital to acknowledge here that navigating stigma terrain requires energy, resources and knowledge capital about cultural value – this is similar to Skeggs and Loveday’s discussion of class struggles and negotiations of valued personhood (Skeggs & Loveday, 2012)—knowing how to play the ‘game’ takes energy and resources (Addison, 2023). Whilst there is value in being hopeful that the sting can be remedied from stigma and people can exist in the world without having their differences stigmatised (Dennis, 2025; Spratt et al., 2025) – it is incumbent on us to examine how and why stigma persistently serves the interests of the most powerful in society and how this is enacted in our day-to-day lives, doing harm to those who are socially vulnerable (Addison and Lhussier, 2025; Tyler, 2020).

In this paper, we frame stigma as relational – informing both structure and agency. We are interested in Tyler’s approach to stigma, whereby stigma violence can lead to a loss of status or decline in a sense of value as a human being – a critical *de*humanisation of the person and/or community (2020). We see this act of dehumanisation aligning with ‘degradations of dignity’, as discussed by Skinns et al (2020), and we are interested in how this impacts a persons’ health and wellbeing (Addison et, al., 2025; NHS Addictions Alliance, 2021). We are alert to how experiences of stigma can contribute to an inverse experience of care (Addison et, al. 2025), which, as Tudor Hart explains in the *Inverse Care Law,* means that the quality *of* and access *to* health*care* varies inversely according to need (Tudor Hart, 1971).

In the remainder of this paper, we will explore experiences of care within police custody through a lens of stigma and from the perspective of detainees.

## Methodology

This study adopted a mixed methods research design which explored standards of healthcare provision in police custody, and included three work packages: ethnography, semi-structured qualitative interviews, and documentary analysis of Police Custody Risk Assessments and Contemporaneous Logs (Rees et, al. 2026, 2026c). The study received ethical approval from Newcastle University Social Sciences ethics board (ID: 13027). This paper focuses on data from the qualitative work package (n-41 in-depth, semi-structured interviews; 35 males, 6 female) and explores the everyday experiences of people who have been previously detained in police custody.

Fieldwork took place in two regions in the north and south of England where two police forces were operational, referred to here as Northton and Sutherland to maintain the confidentiality of participating organisations and interviewees. The constabularies were similar in terms of workforce size and the regions included were both rural and urban areas. In police custody, one Custody Sergeant (CS) is always on shift and will refer a detained person to a HCP with a healthcare concern. It is typical to have one HCP working on a 12-hour shift – however, that HCP might be covering multiple custody suites across a police force area and so is not always directly available onsite. In these scenarios the HCP can provide virtual telephone support from a nearby police custody suite, or the HCP may have to travel to the neighbouring police custody suite if they are needed in-person.

Fieldwork interviews took place between April 2023 to February 2024. Initially, we had planned to undertake interviews within the police custody setting but our access was revoked by the respective police constabularies. As a research team, we reflected on this and agreed that conducting interviews with participants post-custody in the community would provide a less stressful and constrained, and instead more neutral or supportive environment, to allow space and time for participant reflection on experiences. The interviews were conducted face to face, online (via zoom) and via telephone often depending on the preference of the participant. Recruitment was undertaken via gatekeepers within charities aligned with supporting people who were involved with the criminal justice system using a combination of snowball and purposive sampling techniques. Inclusion criteria were limited to self-reported previous experience of the criminal justice system and people aged over 18 years old. Whilst we tried to include a diverse sample of people in our study, this depended on voluntary participation and a predominantly white, male sample pool (see earlier statistics of detainees in police custody as well as our reflections on limitations in the study, HM Government, 2025).

We were mindful that participants who did take part were often dealing with extremely traumatic pasts and present life circumstances so care was paramount in this study. Participants were provided with and talked through an information sheet about the study, and we wanted to ensure that individuals were given space to ask questions to ensure participation was voluntary and undertaken only with informed consent. Participants were also advised that all discussion would be anonymised and identifiers removed. People who are detained in police custody are likely to have experienced trauma and so we were careful in discussions to proceed sensitively—participants were advised that they could stop the discussion at any time and the researcher (SM) was attentive to any signs of distress throughout the interaction and could signpost participants to sources of support if these were needed afterwards. We wanted to ensure that participants understood their rights and our ethical obligations when we advised them that their participation would be treated confidentially (unless immediate harm to self or others was discussed in the interviews) and that they would be given a pseudonym. We advised participants that if we did have to break confidentiality that this communication would be with a support worker not police. We also wanted to make it clear to participants that this work package was not in collaboration with the police and that we were acting as independent academic researchers. To show participants our appreciation and that we valued their time, each person was compensated with a £20 gift voucher.

All interviews were conducted by our study researcher—SM, audio recorded and transcribed. Data were transferred into NVivo, which is a qualitative software that is used to organise and code data with a view to establishing broad categories. We were careful to take a thorough and reflexive approach to analysis: SM read all the transcripts, and a sample of transcripts were read using traditional paper methods by members of the wider team (GR, MB, IM, TF, and MA). We then held a series of analysis meetings where we discussed ideas and a potential coding framework, which was then applied to all data by SM in NVivo, and a smaller sample were coded by different team members. A further set of analysis meetings occurred where we discussed emerging themes from the data. Alongside team meetings, n-8 people who had lived experience (similar to the research participants) were invited and supported via a mental health-based charity in northeast England to become peer analysts in the project and co-analyse excerpts of data. These peer analysts attended 6 analysis meetings facilitated by SM who provided bespoke support so they could learn about the project and receive qualitative analysis training. After these discussions took place a coding framework was agreed by the study team. A thematic approach to analysis was agreed and this was iterative and reflexive (Braun & Clarke, 2006); further analysis meetings occurred within the wider team to discuss inferences, these were regularly reviewed to strengthen the credibility of pertinent themes.

## Findings

In the following section we explore experiences of care within police custody through a lens of stigma and from the perspective of people detained in custody. We focus on three key themes which include i. relational stigma in police custody experienced as judgement, hypervigilance and dehumanisation; ii. stigma harms and inverse care -here we explore harms to health and degradation of dignity through stigma, and finally iii. anti-stigma work in police custody and its positive impacts for health and wellbeing.

Across these themes we are interested in how experiences of stigma are aligned with a degradation of dignity, AAAQ standards, and inverse experiences of quality health*care.*

### Relational stigma in police custody

People detained in custody experience a clear power disadvantage, not least because they have had their liberty suspended for a period of time. For some participants, being in custody heightened sensitivity during social interactions with staff and as such they reported feeling negatively judged for a range of reasons. Tommy describes here how they felt ‘looked down upon’.

I feel like they, they look down at you or something and stuff, you know […] Judgemental. [Tommy, male, Northton]

Elsewhere, Ron shared that they felt they were viewed as ‘scum’ by staff because of their non-local accent and by virtue of being held in custody. This othering process reinforced a sense of dehumanisation amongst participants.

…because you’re in custody, you’re a scumbag anyway. And the accent, up here, doesn’t go, go down well. [Ron, male, Northton]

We also see this stigmatisation in Tony’s experience of custody; to be dehumanised in this way legitimises lapses in respect towards their dignity.

I just find that they come across a bit know-it-all and a bit like, “You’re scum.” sort of thing. You know, they don’t pay any attention to how they talk to people really. [Tony, male, Sutherland]

For our participants, their experience of custody also depended on how their identity and status was perceived by staff. For some, their personal status also compounded experiences of stigma in custody.

This is echoed by Kayleigh who shared insights into her own experiences of custody; rather than being treated as a person who has encountered many health and social challenges, she is treated like a *problem* and thus subjected to stigma because of her drug use. As we can see in Kayleigh’s excerpt below, being labelled as a ‘junkie’ is harmful and functions as a mechanism of stigma, prompting staff impatience and a refusal to recognise a person in pain because of drug withdrawal, and justifying a lack of action.

We’re problems in their eyes, do you know what I mean? ‘Oh, who’s in, another junkie. Fucking hell, what does she want now? She rattling...’ They just don’t give a *fuck*. [Kayleigh, female, Sutherland]

This stigmatisation and dismissal of pain inhibits access to appropriate care; instead, detainees are offered a blanket and hot drink. This minimisation of pain has a negative impact on health and wellbeing—this becomes clear in Kayleigh’s discussion.

‘Cause some of us come in at seven o’clock and some of us come in at eight o’clock, do you know what I mean? No assistance for wellbeing, they just all put it down to ‘they’re just a junkie, they’re not going to die, fuck it, just put them in a cell, they’re fine, give them a blanket and a cup of coffee. [Kayleigh, female, Sutherland]

Whilst basic needs are met, this minimum standard of treatment feels degrading to Kayleigh, rather than respectful. Dehumanisation was also evident through refusal of minimum standards of respect for an individual’s personhood, which we align here with a degradation of dignity. Carl shares how case logs attached to previous incidents and linked to his name immediately shape how staff treat him according to an implicit hierarchy of offences.

They get my name and straight away there’s a warning on my name and that’s it, they completely switch direction, whether they were treating me nice or whatever. And when they get my name and the markers on my name, that’s it. They completely treat me like I’m not even a human being. […] Just easy targets. They’ve got all these other people, violent criminals, stuff like that, that they’re scared to deal with, so when they get someone who’s unable to defend themselves, and stuff like that, they mistreat them, because of all this stuff that goes on with the other ones that they can’t take it out on them. [Carl, male, Sutherland]

The treatment Carl receives from detention officers may be indicative of broader structural problems in police custody—work pressure, high risk, and staffing levels may contribute to these types of poor interactions whereby frustrations are vented on certain detainees. Stigmatisation may not always be conscious or intentional. What can appear to be ‘banter’ or ‘fun’ to staff in their interactions with detained people can be experienced as a degradation of dignity.^2^In this instance, Carl shares how he is treated like entertainment for the staff when he regains consciousness in his custody cell. For Carl, this is experienced specifically as a lack of ‘care’.

A lot of the time, when I do come round, I didn’t even know where I am. But they don’t care. Basically, they treat me as a bit of a joke when I get in there. I’m their little bit of entertainment, a lot of the time. [Carl, male, Sutherland]

Dehumanisation can also be inflicted in other ways, for example, some participants felt they were treated as just a number – this experience of objectification can feel like a visceral denial of personhood (*‘treat you like shit’*)*.*

They just treat you like shit. Like, they just think you're fucking like, basically what you’re classed as in the police station, you’re just classed as a number. Because of your number cell. [Tyler, male, Northton]

These effects of stigma are even more observable in Gordon’s account where they describe being strip-searched in police custody. They were made to feel embarrassed and humiliated because of the actions of the custody staff who attempted to imply Gordon had a strong body odour. This contravenes the directive to maintain human dignity.

They strip-searched me and then tried to… They try to humiliate and embarrass you. I’m like, “I’m not embarrassed, you’re the one that should be embarrassed.” Trying to spray where they were strip-searching me as if to say, “Oh, it smells.” (makes noise) [Gordon, male, Northton]

This stigma mechanism focused on the body as abject (Tyler, 2013), but this attempt to degrade dignity and dehumanise him was resisted by Gordon. Attempts to resist and subvert ‘power trips’ from custody staff were echoed elsewhere by participants. Like Gordon above, Tony comments on some of his interactions with custody staff – perceiving them to be rude and condescending.

…you don’t have to be like on a power trip, as I call it. It comes across to me as they’re quite condescending in how they come across. They’re quite rude sometimes… [Tony, male, Sutherland]

Ravi highlights how interactions with custody and police staff can become fraught as a result of power imbalances. Implicit in this excerpt is a sense that detainees are not treated with dignity and this is marked by a lapse in respectful communication.

I call some police officers, they get bouncer syndrome. So they have what I call the yellow armband on their arm, and all of a sudden, they’re in a position of power and they can talk to people how they want. I sometimes think the genuine message of what a police officer is there to do, protect and serve, is lost in translation. [Ravi, male, Sutherland]

Whilst detainees in our study reported feeling dehumanised and degraded by custody staff, there were occasions when this mechanism of stigma was weaponised by detainees to retrospectively describe custody staff, as Gordon shows:

…they’re just obviously inbred fucks, aren’t they? [Gordon, male, Northton]

### Stigma harms and inverse care

In this section we explore harms to health and degradation of dignity through stigma. We also discuss the ways in which detained people navigated inverse care experiences and this stigma landscape.

Feeling stigmatised, unseen and unheard has significant negative impacts on how and when people speak up and ask for help with a health-related issue (Addison., 2023; Metzl & Hansen, 2014; Muncan et al., 2020). This was evident in our discussions with detained people in custody. Participants shared that how they were treated by staff affected whether they would be likely to disclose health related challenges. Natasha gives us an insight into the many voices that have gone unheard and the missed opportunities to engage with a marginalised population experiencing health problems.

…the amount of people then that could have spoken or want to speak, it’s just they’re… It’s like they’re putting a brick wall in the way of it. It doesn’t matter how much you’ve done, if you’re still in need of help, do you know what I mean? You’re still worthy of that. [Natasha, female, Sutherland]

The design of police custody and processing procedures did not foster a sense of ‘safety’ or trust in staff, both of which are foundational to enabling health disclosures in any health-related setting. As shared by Ravi:

I feel like for such an intimate moment where you’re handing over your possessions, that shouldn’t be done in public. That shouldn’t be done in front of people. That’s something that’s quite an intimate moment, and you’ve got to remember, that’s the last moment you get before you're locked away into a confined space. So, “Yeah, okay. Look, I haven’t had my meds. This is what I’m on. I’m really struggling,” and it was. I didn’t have the tools at the time to say that. I didn’t feel brave enough to say that. [Ravi, male, Sutherland]

Some participants felt like their health needs were not taken seriously because they were stigmatised by staff members as ‘problems’. This individualising rhetoric is commonly directed towards people who use drugs and alcohol, and used to blame and shame individuals for their addictions instead of providing support (Addison et, al. 2022; Addison, 2023).

So unless you’re dying, there’s no-one coming for you. To be honest, even if you was like that, they’d just call an ambulance, like, it’s really bad. They just see addicts as, as problems, and they all ask the same fucking things, they don’t want to give out any medication, um, yeah, and they just, they’ve got no compassion, they’re just, they see, yeah, everyone’s just treated the same, like shit. [Gordon, male, Northton]

Elsewhere, some participants shared a reticence about disclosing mental health conditions with custody staff because of a perceived sense of stigma attached to mental health. For Felix, there was a lack of understanding amongst staff about mental health more broadly.

If the police could understand mental health, not just stigmatise it, “Oh, you’ve got mental health problems-” honestly, they need to understand the full spectrum. [Felix, male, Sutherland]

Others were concerned that any health disclosures would stigmatise them and compound disadvantage even further and may even negatively impact their case.

I feel that if you say that you’ve got serious mental health problems they sort of put you in a category of people that don’t know what you’re doing or they think you’re under the influence or they think that they can be demeaning in some form or another. I don’t think they take mental health very seriously at all. [Tony, male, Sutherland]

The custody environment is a stressful place to be, especially for detained people with co-occurring health conditions and neurodiverse needs. Asking for help can be incredibly difficult for some people in this context, this is made harder due to a sensitivity to stigma of being seen as a burden or a nuisance. Tony tells us about his experience in custody and how asking for help was framed as asking for ‘extra’, in other words—more than what could be considered ‘basic needs’. This is indicative of a health need being converted into an ‘unmet need’.

…it heightens your emotions, it puts you on edge, it can make what problems you’ve already got a lot worse than what they already are in the beginning, you can feel not- I don’t know how to put it, it’s not discriminated against but it makes you feel like you’re different because you’re asking for extra help. Sometimes it feels like you’re a burden asking for the extra things from them. [Tony, male, Sutherland]

Rees research with health care practitioners revealed that staff members found ways to synthesise their medical and legal responsibilities to attend to both care and risk (Rees, 2023). Our data also echoes this finding to an extent – HCPs provided care through a bio-medical model of health which minimised risk. However, the experience of this form of ‘care’ was quite detrimental to the detained person, as Tony recounts:

People with mental health, they will talk to you about it but they’re mostly concerned with, “Are you thinking about killing yourself, are you in breach of custody?” More covering their backsides, do you know what I mean, rather than actually identifying what your issues are and actually putting the right care in place for you while you’re in their protection. [Tony, male, Sutherland]

This is revealing of the inverse care law operating in custody – quality of care varies inversely to need (Addison, et, al. 2025; The Health Foundation, 2022; The Lancet, 2021): whilst care may meet the minimum threshold required through PACE Code C (HM Government, 2019), a bio-medical model cannot capture information regarding the wider social determinants of health nor does it account for quality of care set out in the AAAQ standards by the World Health Organisation. We can see these impacts in Ron’s experience of custody and how interactions devoid of care can be detrimental to trust and engagement. In this instance, Ron experiences being treated as ostensibly criminal and thus stigmatised. Understandably, he wants to leave this space as soon as possible and this can lead to missed opportunities for health engagement (Addison et, al. 2025).

They just don’t show nothing, they just should have some more care, you know, just ‘cause you’re a criminal, or you haven’t even been found guilty of anything yet but you’re getting treated like a criminal straight away, so all you want to do is get out of that fucking cop shop as soon as you can. [Ron, male, Northton]

This sense of emotional numbness amongst professionals is described by Ron and experienced as a lack of compassion. However, there can be very good reasons that professionals working in highly pressurised and emotionally heightened roles may manage and suppress emotional behaviour and body language, not least to protect against burnout and compassion fatigue (Hochschild, 1983; Rees, 2023).

…these are cold people, you, you, you know, there’s no caring NHS or something, if you’ve got an NHS badge on there, then why are you so fucking cold? And these are intelligent nurses, supposed to be. [Ron, male, Northton]

In Gordon’s account, they share their experience of people they report to be medical staff. In this excerpt, quality of care becomes central and is expected as part of their job role:

As soon as they know you’re into heroin or have done anything like that, straight away, they become like… I’m not stupid, I see the demeanour change and their… I’m not stupid, I can see them change. It’s just like… I won’t put up with that. It’s like, “You’ve got a job to do, do it.” [Gordon, male, Northton]

We see elsewhere in Gordon’s account that there is a sense of injustice regarding treatment in custody and a need to be treated like a human being.

Again, even if you are guilty, you’re still a human being, shouldn’t you still be treated with… I mean, you know, there are some nasty people out there. Like I say, they’ve got very difficult jobs to do and I understand that. I’m not stupid, I understand, however, that’s still no excuse. [Gordon, male, Northton]

Navigating stigma and retaining dignity in a custody space was not easy for participants. Kyle reveals the level of persistence needed to gain the attention of custody staff and HCPs.

I’ve known a lot of heroin and crack users. I’ve never known them not to be like that. Especially, the ones who have been arrested so many times, they know all the tricks and whatever. They may know you’ve got to keep on at people to get noticed. If you say it once, “Can I have this?” You’re not going to get it. [Kyle, male, Sutherland]

This is also complicated by having to present as ‘authentic’ and trustworthy to get health needs addressed.

From what I can remember, you do ask for it for quite a while. You do have to keep on and say, “I need to see a doctor, I’m rattling.” […] I think they do but it does take a while. I tell them I need to see a doctor, I’m going to fucking fit in a minute, do you know what I mean. I remember it being quite, like, I really thought I was going to fucking go into a fit until when I seen the doctor. And when I’d seen the doctor, I remember it being quite quick […] they only gave me one tablet and I wanted two. I remember that. I said, “I’m not the usual alcoholic.” I was, like, “My intake is a lot more higher than anyone else’s.” I said, “One tablet’s going to do F all.” [Kyle, male, Sutherland]

This activity requires a high level of cultural health capital (CHC) – that is ‘knowhow’, to get health needs recognised and met (Chang et al., 2016; Dubbin et al., 2013).

### Anti-stigma work

In this final section we highlight the importance of compassion and dignity in these interactions in police custody. Compassion and the maintenance of dignity can have a positive impact on a detained person’s experience of custody, and these are key components of anti-stigma work more broadly (Anti Stigma Network, 2026).

…the people that are working there, they need to know the score and understand and be able to connect with the, um, you know, like, the inmates […] I’d say, and maybe just a bit more compassion rather than judgement. [Kayleigh, female, Sutherland]

…if they treated everyone on the same sort of path, or had a better understanding about the stuff that everyone is going through at the moment, like now the cost of living, it is hard. Yeah? So if I was a junkie or a crackhead, I’d be going out there robbing for my habit, because obviously I’m not going ‘to have much money. I know it sounds like it’s a farce but obviously they should see it that way, and still treat somebody as humanely as possible. [Felix, male, Sutherland]

This work would help to reduce a detained person’s anxieties about perceived stigma and to feel less judged in the custody setting.

Some participants also highlighted the need for better understanding from staff regarding wider social determinants of health. Felix identifies the need for education about the causes of ‘risky behaviours’ like drug use and associated criminogenic factors. He felt that if this was improved it might also positively impact social interactions and treatment of detained people in custody.

They also need to be schooled in mental health, alcoholism, drug abuse, because all these things are all, kind of, like, interconnected. So if someone has had something bad go on, which has resulted in them being a heroin addict, and a crackhead, or whatever, that’s their fight or flight. So, because you’re known as that category of person, automatically the police are going to treat you a certain way. [Felix, male, Sutherland]

Whilst compassion and maintenance of dignity for detained people in custody was central for many of our participants, some participants also showed compassion for custody staff and recognised that it was a difficult job to do.

…when you do start getting aggro-ed and irate, then they obviously are going to be a bit rougher with you. So I have been roughed up or whatever you want to call it, but nothing due to their fault, it’s been my own fault. So realistically, they’ve never done anything bad or wrong, it’s been my own fault. They’ve always been pretty good with me anyway. [Kieran, male, Northton]

They probably get annoying drunks all the time, annoying druggies, like, “I want this and I want that, I want this, I want that. No, that’s not enough. Fuck you.” So they’re probably just sick of it, you know what I mean. If someone talks to you shit all day you’re not going to be fucking happy. [Kyle, male, Sutherland]

Participants wanted to be treated like any other person (albeit without their liberty), and this meant with dignity and respect – this is fundamental to AAAQ standards. Professional practice, quality care and acts of compassion can have very positive impacts on the lives of detained people, even if it is not obvious at the time. Alistair shares with us a life-changing interaction with a police officer who displayed these anti-stigmatising qualities (respect for dignity, quality care) regarding his suicide attempt.

The police officer who arrested me when I jumped off the bridge took me to […] hospital. He was sound. He kept saying, “You need help. You need help.” He was the one who arrested me when I was fighting with my mate, and he knew a bit about me. He said, “Come on, Alistair, you need this. You’ll be dead if you don’t do this.” […] Then, when he went out of the hospital, he just patted me on the back and said, “Come on, you need this. Get on with it and do it. Yeah, a human being, instead of a fucking criminal or a baghead. Yeah, he was sound. He gave me a cuddle and that […] I wouldn’t mind thanking him, because without him, I’d have just fucking gone out and done it again. I really would. [Alistair, male, Northton]

The quality of ‘care’ that a person receives matters and can have immeasurable positive impacts on a person’s life. Anti-stigma work in police custody is important and foregrounds the need to treat detained people as human beings deserving of respect, compassion and dignity. This work may help to improve health engagement and avoid missed opportunities to offer someone support with a health-related issue. Unfortunately, the current structural limitations of police custody, specifically the understaffing of DOs and HCPs, serves to reproduce rather than reduce stigma.

## Discussion

This paper is revealing of the lived experiences of people detained in police custody, a neglected population in health research, and is part of a qualitative work package nested within a larger mixed-methods study (see Rees et, al. 2026c for in-depth discussion). Our analysis has explored harms to health and degradation of dignity generated through stigma from the perspective of people detained in police custody. We focused on three key themes which included i. relational stigma in police custody experienced as judgement and dehumanisation; ii. stigma harms and experiences of ‘care’, and finally iii. anti-stigma work in police custody and its positive impacts for health and wellbeing. Across these themes we were interested in how experiences of stigma aligned with a degradation of dignity, AAAQ standards and inverse experiences of quality healthcare*.*

Our findings have shown that a number of detainees experienced stigma whilst in police custody for reasons which coalesced around identity characteristics, social status, drug and alcohol use, mental health, presumed guilt, bodily smell, and frequent healthcare requests. This analysis revealed a process of ‘othering’ – to frame someone as ‘unhuman’, ‘scum’ or ‘junk’- and this mechanism of stigma served to erase personhood and compound power differences between detainees and custody staff (Tyler, 2013, 2015). Participants reported feeling hyper-vigilant to this sort of interaction with staff; according to Tyler, being treated like ‘scum’ is akin to identifying someone as a ‘wasted human’ (Tyler, 2013). Added to this, being treated simply as a number also had a dehumanising effect on the people in our study. As Tyler writes (2020) this mechanism of stigma is anchored in many historical examples – it has previously been used to reduce someone to ‘chattel’ and was used to identify slaves, account for property, and for prisoners of war. This ‘de-naming’ and experience of being treated as a number occurs in prisons and other custodial spaces today; this numbering process can be used to indicate a significant change in human ‘liberty’ status and make order and control easier.

In our analysis, participants were also sensitive to a custody culture which presumed guilt and negatively shaped staff-detainee interactions. As such, perceived ‘guilt’, and the stigma attached to this, became an operative assumption amongst staff until this was disconfirmed or the person was released from custody. This presumption of guilt was particularly ingrained in a place of custody where custody staff were exposed to a higher proportion of people who presented with ‘risk factors’ (Klockers, 1980), (e.g. alcohol and drug use). In this context, our analysis shows that stigma and degradation of dignity can appear justified to staff when anchored in presumed guilt. These kinds of interactions had a dehumanising impact on our participants. and were aligned with harms to their health and wellbeing.

Detained people in custody also discussed the strategies that they employed to navigate stigma in police custody. Our participants were not passive to the harms of stigma; some of our data showed that the detained body was treated as ‘abject’ (Tyler, 2013) and regarded as smelly, leaky and dirty by some custody staff. However, these attempts to stigmatise and degrade human dignity were resisted by participants. One participant vocally called out the unfair treatment they received from staff during a strip search and turned a reproachful lens back on the custody staff who were attempting to dehumanise him. There were also moments where stigma was weaponised by detained people and used to retrospectively attack custody staff (one participant described staff as ‘in-bred’). It is important to acknowledge the power imbalance here in these dynamics, whereby detained people have more at stake if they partake in combative interactions with staff, nevertheless, detainees navigated this by ‘talking back’ to detention officers within the privacy of their own internal dialogue.

Whilst minimum standards of care detailed in PACE Code C (1984) are paramount, these can often be addressed using a bio-medical model of care with a view to minimising risk. We argue that this approach to care can lead to dehumanising and stigmatising experiences by detained people. In one example, the offer of a cup of coffee (instead of appropriate healthcare) revealed a dismissal of pain caused by drug withdrawal. The minimisation of health needs was a frequent occurrence for our participants. This presents an interesting comparison to Skinns et, al.’s findings whereby the offer of a cup of tea and a chat helped to centre a detained person in discussion and respect their dignity whilst in custody (Skinns et al., 2024). In our study however, participants felt dismissed and ‘fobbed off’ with a cup of coffee in lieu of good quality care. Our analysis shows that the context in which the gesture is offered is very important, on the one hand it can reveal person centred care (Skinns et, al’ study) and on the other hand this gesture can serve to dismiss pain and minimise care provision (our study). We suggest to policymakers, commissioners and healthcare providers in police custody that, rather than a bio-medical approach to ‘care’, what is needed is a relational and more holistic approach set out in AAAQ standards, which recognises a person’s ‘humanness’, provides quality care, and maintains a person’s dignity.

Our analysis also shows that stigma had a chilling effect on levels of engagement by detained persons with staff regarding health-related issues. In some cases, participants felt that they were considered untrustworthy and disbelieved by staff when asking for help, this tension revealed that participants had to ‘know how’ to get their needs met – this knowledge of how to navigate service provider interactions, especially when there is a power imbalance, has been conceptualised elsewhere as cultural health capital (CHC) by Chang et, al (Chang et al., 2016). Even when our participants were persistent and used CHC to get their needs met, they felt that HCPs lacked compassion and presented as unfeeling, and this contributed to feeling dehumanised in their interactions. We noted that feeling stigmatised, unseen and unheard like this had significant negative impacts on how and when participants felt able to speak up and ask for help with a health-related issue (Addison., 2023; Metzl & Hansen, 2014; Muncan et al., 2020). Furthermore, many of our participants told us that they did not disclose major and minor health conditions, particularly related to their mental health, because they did not feel that detention officers had the adequate skills or resources to offer quality support, nor did they feel they were in a safe space which afforded them privacy, respect and ‘care’. Further analysis shows that these encounters exemplify the harms arising from stigma power and represent many missed opportunities to engage holistically with under-heard and under-served people experiencing health problems in police custody (Hatzenbuehler et al., 2013; Link et al., 2017); although it should also be remembered that many people also provide extensive and detailed medical health histories during booking-in risk assessments, which are often ignored or deemed to be acts of manipulation by Custody Sergeants until corroborated by alternative means (for instance prior police records). We argue that there is an urgent need to research and understand the contextual factors around social and health inequality outside of custody and how they are interconnected with reasons for being detained in police custody, and the quality of care needs to be of a higher relational quality outlined in the AAAQ standards to help participants feel respected and safe enough to disclose their health needs.

We also found that the foregrounding of risk management and a bio-medical model of ‘care’ in these interactions can potentially lead to a ‘care’ vacuum within healthcare provision in police custody, leaving no time or space to maintain a person’s dignity and personhood. This finding illuminates aspects of the inverse care law in action – where care varies inversely to need (Addison et, al. 2017, 2025). Our analysis shows that by only attending to bio-medical markers of health, it is not possible to capture information regarding the wider social determinants of health nor does it account for quality of care experienced by detainees at a relational level (Addison et, al. 2023). This is where stigma can be felt under the skin and have serious consequences for levels of health service engagement, disclosure, and outcomes (Addison et, al.., 2025; Marmot, 2018; The Health Foundation, 2022; The Lancet, 2021; Tudor Hart, 1971). This care vacuum has negative implications for meeting AAAQ standards and maintaining human dignity, and should be prioritised by policymakers, commissioners, and healthcare providers in custody.

Finally, the importance of compassion, and the maintenance of dignity cannot be underestimated in interactions with detained people and their treatment for health-related issues. Compassion and an understanding of social inequality were key to a less stigmatising experience of police custody.

### Limitations of the study

This study has many strengths, especially in its use of a mixed method multi-phase approach across two different geographical sites. However, our study has some limitations. In 2024, the Home Office reported that 84% of people detained in custody in England and Wales were male, and 78% were of a white ethnic background. As such, our sample reflects this and is largely white and male, although does include some representation of voices from minoritised communities. To address this, we would need to adopt a different sampling approach weighted towards minoritised participants in future research. Access to police custody was initially granted by police partners at the outset of the project but then this permission was revoked due to additional police restrictions. This prevented us from conducting interviews with detained people whilst in police custody; instead, we undertook fieldwork in the community (3rd sector) and so participant experiences are based on their recollections and reflections of events in custody. Undertaking fieldwork in this way had the added benefits of avoiding selection bias (police identifying potential participants) and helped to preserve anonymity and confidentiality on behalf of participants. Having time to reflect on experiences also meant that participants were able to discuss any consequences of detention post release. Trusted support workers were also available in 3rd sector organisations to support participants if needed; this support would not have been available in a police custody setting.

## Conclusions and recommendations

There are significant challenges present for staff and detained persons in police custody which can make interactions fraught. It is a highly pressurised environment where a power imbalance is necessitated between staff and detained people in custody because of the removal of liberty. However, this can lead to lapses in care and respect for a detained person’s dignity – experienced by and through stigma. Detainees in police custody are a neglected population group in health research – and yet what happens in this setting has the potential to lead to widening health inequalities and negative ramifications on health and social care systems in the community. We have brought together different bodies of literature, alongside a stigma theoretical framework to help show that the impact of stigma on the quality of healthcare and maintenance of dignity in police custody settings is predominantly negative.

However, there are learning opportunities here for policymakers, commissioners, academics, police, and healthcare providers that will help reduce the presence and impact of stigma on people detained in custody and facilitate a more positive experience of quality healthcare if we utilise the AAAQ standards. As part of a wider programme of work focused on restructuring police custody (Rees et, al. 2026b, 2026c), we recommend to stakeholders to pilot an anti-stigma training programme in a sample of police custody settings to address health inequities across delivery and provision of good quality health care, and to evaluate the feasibility and implementation of a national roll out of anti-stigma activity. At an individual level this could include an anti-stigma education intervention which incorporates the AAAQ standards (Stangl et al., 2019) for detention and custody sergeants, as well as HCPs. Whilst we are not claiming that anti-stigma training is a panacea, it has several potential benefits: i. it has the potential to increase compassion, ‘professional curiosity’, and understanding amongst staff working in custody regarding the complex lives of detained people, ii. it can raise awareness of and encourage reflection on stigma practices in a custody setting, and how these can be harmful to health and wellbeing, iii. it can help custody staff to understand how vital it is to treat detained people with dignity, respect and to acknowledge ‘humanness’.

At a structural level, anti-stigma work could support policy and guidance reform within custody; it could lead to the coproduction of the National Strategy for Police Custody between police and practitioners, people with lived experience and academics; as well as greater investment in HCPs embedded in custody settings by healthcare providers to ensure quality care can be provided (Rees et, al. 2026a, 2026b, 2026c).

What happens within police custody has a ripple effect on a person’s life once they are released so we need to take seriously the impacts of stigma and how this harms health, impacts engagement with health services, and widens inequalities. In this regard, the provision of good quality healthcare in police custody, that is free from stigma, is vital and should be prioritised by policymakers, commissioners, the police, and healthcare providers and practitioners operating in police custody.

## Data Availability

Due to the data sharing agreements between the two police forces and Newcastle University, data cannot be shared beyond the research team. We have uploaded our data analysis tools, especially our conceptual mapping to the UK Data Service Archive.

## Footnotes

1 Six strategic principles: 1. Justice and the protection of the public; 2. Detention in police custody is safe, ethical and used only when appropriate; 3. Custody is non-discriminatory and transparent; 4. Everyone is treated with dignity and respect; 5. Custody practitioners are professional experts in their field; 6. We will continually learn, improve and innovate.

2 Readers may be interested in the BBC documentary ‘Undercover in the Police’ which explores these issues: https://www.bbc.co.uk/programmes/m002k7k6
